# Influence of Cardoon Flower (*Cynara cardunculus* L.) and Flock Lactation Stage in PDO Serra da Estrela Cheese

**DOI:** 10.3390/foods9040386

**Published:** 2020-03-26

**Authors:** Élia Fogeiro, Paulo Barracosa, Jorge Oliveira, Dulcineia F. Wessel

**Affiliations:** 1 School of Agriculture, Polytechnic Institute of Viseu, Quinta da Alagoa – Estrada de Nelas, Ranhados, 3500-606 Viseu, Portugal; eliafogeiro@gmail.com (E.F.); pbarracosa@esav.ipv.pt (P.B.); joliveira@esav.ipv.pt (J.O.); 2 CITAB, University of Trás-os-Montes and Alto Douro, 5001-801 Vila Real, Portugal; 3 QOPNA & LAQV-REQUIMTE, Department of Chemistry, University of Aveiro, 3810-193 Aveiro, Portugal

**Keywords:** Serra da Estrela PDO cheese, *Cynara cardunculus* L., ewe’s milk, physicochemical characteristics

## Abstract

Serra da Estrela (SE) cheese is one of the most appreciated Portuguese cheeses, being produced only from raw ewe’s milk, cardoon flower and salt. Cardoon takes part in two important processes in cheese production—coagulation and proteolysis—contributing to its unique features. Furthermore, milk chemical characteristics change during the milking season, being another factor that account for the high variability of cheese attributes. Therefore, the purpose of this work is to study the influence of cardoon flower (commercial, 6 M and 3 M) and flock lactation stage (November 2018, February and April 2019) in the final characteristics of SE cheese. The parameters analysed were moisture, protein, fat and salt contents, texture and colour. Results showed that flock lactation stage has the highest influence in all the studied characteristics, corresponding the early stages of lactation to the most protein-rich and low-fat cheeses. Cardoon flower affects mainly fat and rind colour. This study allows us to conclude that seasonal changes in ewe’s milk have a considerable impact in cheese attributes, and that although cardoon type had a more restrained effect, when used with expertise it may help adjust cheese sensory characteristics in order to obtain a final product that matches consumer acceptability requirements.

## 1. Introduction

Serra da Estrela (SE) cheese is a Portuguese traditional agro-food product with Protected Designation of Origin (PDO), manufactured only from raw ewe’s milk (exclusively from Serra da Estrela sheep), cardoon flower (*Cynara cardunculus* L.) and salt [1,2,3]. SE cheese is perceived as a unique high-quality food product with great economic importance [4,5], highly appreciated for its strong aroma, slight bitterness and semi-soft texture [6]. The cheese final properties are dependent on a variety of parameters, including the characteristics of used ingredients and processing factors. Ewe’s milk production is seasonal, and even the largest ewe’s cheese factories interrupt production during summer months [7,8].

The chemical composition of bulk milk changes during the milking season, particularly in terms of fat and protein content, being influenced by ewe’s feed (natural pastures or commercial feed) and the flock lactation stage [7,9]. Therefore, variation in the nutritional composition of milk is expected to affect the final characteristics of the cheese [8].

The cardoon flower is traditionally used in the Mediterranean basin, in the manufacture of goat and ewe’s cheeses, as a mandatory coagulant for production of several artisanal PDO cheeses [10,11,12]. Ewe’s cheese produced with cardoon extract is recognised as a high-quality food product with soft paste and an enhanced flavour that is slightly bitter and piquant [12,13,14]. The milk-clotting activity of cardoon flower is due mainly to the activity of cardosins A and B [15], two aspartic proteases similar in activity and specificity to chymosin and pepsin, respectively [16,17]. The cardoon flower activity depends on numerous factors such as the cardoon flower genotype [10,18,19], the part of the flower used [20,21], its stage of physiological maturity and the flower drying time [12,21].

Coagulation of milk is a crucial step, which involves the enzyme-mediated cleavage of k-casein at the peptide bond Phe105-Met106 [22], leading to casein micelles destabilisation and causing aggregation that yields a clot and a gel afterwards [23]. In addition, milk-clotting enzymes usually possess a broader proteolytic activity towards α_s_- and β-caseins at much lower rates than with k-casein [22,23,24]. The continuous hydrolysis of caseins by a vestigial coagulant—proteolysis—has a decisive role in the initiation of cheese ripening, since it produces substrates for flavour development by bacterial microflora [25,26]. Therefore, coagulation agents play a determinant role in the definition of curd characteristics by influencing micellar aggregation, gel firming speed and the final gel firmness, which may influence cheese moisture content, texture and flavour [27]. As seen, cardoon flower type and ewe’s milk quality, which vary according to milk production month, are two factors that can significantly affect cheese characteristics.

As far as is known, this is the first study in the field to investigate the effect of cardoon type and production month, both individually and simultaneously, in physicochemical properties of SE cheese. Furthermore, special care was taken to assure that the effects of other potential sources of variation such as ewe’s milk producer and the different techniques used by the manufacturers were minimised.

In the present work, two studied cardoon flower types, 3 M and 6 M, and a commercial cardoon flower used by the producer in the cheese factory daily work, were used to manufacture SE cheese. The biochemical characteristics of six cardoon flower types, namely its cardosin profiles, have been studied by Barracosa et al. [10]. From the range of cardoon flowers investigated, 3 M and 6 M were selected to be used in this study, since they present very distinct cardosin profiles. 3 M cardoon type presents an ion-exchange chromatographic profile with predominance of a cardosin A1 peak and absence of a cardosin A peak. On the other hand, 6 M cardoon showed a clear prevalence of a cardosin A peak. These differences found in cardosin profiles may influence the gelling properties of the coagulant, thus influencing cheese attributes [23].

Therefore, the purpose of the present work was to study the influence of cardoon flower (6 M, 3 M [10] and commercial) and cheese production month (November 2018, February and April 2019) in the final characteristics of PDO SE cheese.

## 2. Materials and Methods

### 2.1. Cheese Manufacture and Sampling

The PDO SE cheese was manufactured by Queijaria Quinta de São Cosme (Vila Nova de Tázem), a certified cheese producer from the PDO region, according to their regular manufacture procedure, reported in Caderno de Especificações do Queijo Serra da Estrela [28]. The milk was always obtained from the same producer.

Cardoon flowers of different types were used for the coagulation: (i) commercial cardoon, commonly used by the producer, (ii) 6 M cardoon, rich in cardosin A and (iii) 3 M cardoon, with no cardosin A [10], giving rise to three independent batches each with 30 L of milk. Cardoon flower was used as an extract, prepared by mixing cardoon flower previously dried and macerated with about 200 mL of water. It was used 0.4 g of commercial cardoon and 0.2 g of 3 M and 6 M cardoons per litre of milk.

Cheese yield was calculated by the ratio of cheese weight (kg) to milk volume (L). It was determined by weighing each batch of cheese after the pressing process, in day 1. The average yield of cheeses produced for this study was 0.241 kg/L.

Cheese weight loss was calculated by:

(1) $$Weight\;loss\;\left(\%\right)=\frac{W_{38}-W_{1}\;}{W_{1}}*100$$

where *W*_1_ and *W*_38_ are the cheese weight at day 1 and 38, respectively.

The cheeses were produced in November 2018, February and April 2019 and maturated for 38 days, being ready for analysis in January, March and June 2019, respectively.

For this research, three cheeses of about 0.5 kg each were chosen randomly from each batch, in each month, and for physicochemical parameters triplicates were done. Milk samples from each cheese production were previously collected and analysed.

### 2.2. Colour

Cheese colour parameters obtained from rind and paste were measured after the maturation period. A Chroma Meter CR-400 (Konica Minolta, Chroma Meter CR-400, Tokyo, Japan), calibrated with a white standard tile, was used to assess the CIE Lab colour space coordinates, L* (lightness), a* (green-red) and b* (blue-yellow) values.

The rind colour was determined both in the bottom and top faces of the cheese. Paste colour was measured by removing previously 1 cm of cheese rind.

### 2.3. Texture

Textural properties were evaluated using a TA.XT Plus texture analyser from Stable Micro Systems (Godalming, Surrey, UK). Texture Profile Analysis (TPA) was performed using a P/1S spherical probe to assess hardness, adhesiveness, springiness, cohesiveness and resilience. The operational parameters were: pre-test speed = 3.00 mm/s, test speed = 1.00 mm/s and post-test speed = 1.00 mm/s; compression distance= 4.00 mm; trigger force = 0.049 N; and load cell = 50 kg. Cheeses were stored at room temperature one day before analysis. The measurements were carried out in both top and bottom sides of the cheese.

### 2.4. Chemical Analysis

Total fat, total protein, salt and moisture content were assessed through Fourier-transform near infrared spectroscopy technology, using a NIR Master™ FT-NIR spectrometer (Büchi, Flawil, Switzerland).

### 2.5. Statistical Analysis

To analyse the influence of cardoon flower type, milk production month and their interaction, the data obtained was subject to a two-way analysis of variance (ANOVA) with a bi-factorial model. Differences of means were analysed with Tukey’s post hoc tests. The mean squares were extracted from the ANOVA tables and the partial variance concerning the studied factors (cardoon flower type, flock lactation stage and their interaction) and the residues were calculated, giving the relative influence of each of the factors in the studied parameters. The IBM SPSS Statistics software for Windows version 25 (IBM Corp., Armonk, NY, USA) was used for data processing and statistical analysis, with a level of significance (α) of 0.05.

## 3. Results and Discussion

### 3.1. Milk

Results described in Table 1 show that during the milking season, protein content remained approximately constant, whereas fat increased from November to February, continuing to be stable from thereon. The values obtained are in line with previous studies for SE sheep milk [29,30]. Several other studies show an increasing tendency in fat and protein content during the lactation period [31,32,33,34,35].

The milk samples analysed showed higher acidity values than the ones reported in a previous study [29]; however, the values are in line with those recommended for SE PDO cheese [36].

### 3.2. Cheese

Table 2 shows the statistical significance of flock lactation stage, cardoon type and their interaction on the final characteristics of PDO SE cheese. Flock lactation stage is understood in this work as the cheese production month.

Cheese production month has a very significant influence in most of the studied characteristics, in contrast with the cardoon type, whose effect is generally non-significant.

Cardoon type affects mainly fat concentration and rind colour parameters, whereas cheese production month influences also rind texture and paste colour. Moreover, from all the studied attributes, rind texture characteristics stand out by being highly influenced (>90%) by cheese production month.

Significant interactions between cheese production month and cardoon type were detected for rind colour coordinates L*, b*, ∆E; paste colour coordinate a*, cohesiveness, resilience, fat and protein.

In the following sections, the individual effect of each of the studied factors, such as cardoon type and production month, was determined by analysis of variance.

### 3.2.1. Influence of Cardoon Type

Although studies regarding SE cheese texture evaluation have already been conducted, this work was planned to allow the control of factors liable of bringing variation to the results. Thus, the milk used in this study comes from only one producer and the cheese was manufactured in the same cheese factory, in all months of production.

The analysis of variance, whose results are displayed in Table 3, identified the significant (*p* < 0.05) effect of commercial cardoon in a* and b* colour coordinates of cheese rind. The use of commercial cardoon in cheese manufacture led to the production of yellower cheeses than the ones produced with the other cardoon types studied. These data suggest that cheeses produced with different cardoon types may be distinguished through visual assessment by consumers. Concerning cheese paste, 6 M cardoon stands out from the rest by presenting a tone closer to neutral in the a* colour coordinate.

Cardoon type had no statistically significant effect in cheese texture. However, there was a clear tendency for cheeses produced with commercial cardoon to present lower values for rind hardness. The diversity of cardosin profiles of each cardoon used in this research may help explain some of these differences. The coagulant is the main factor responsible for primary proteolysis and it is believed to cause softening of cheese in the initial stages of ripening, via disruption of its three-dimensional protein matrix [37]. Serra da Estrela cheese is expected to have a semi-soft texture [6,38], an attribute highly valued by the consumers. The results obtained can be used to produce cheese with characteristics closer to consumer’s preferences. Furthermore, the variation of the cheese texture also influences the release of aroma compounds during mastication [37]; therefore, it is able to influence the flavour perception.

Concerning chemical properties, cheeses produced with 3 M cardoon showed the highest protein percentage, while those produced with 6 M cardoon displayed the highest fat content. Fat and protein are determinant for the development of cheese final attributes. Fat is a provider of desirable sensory characteristics, besides having an important role in cheese texture. Changes in fat content can influence the cheese matrix by altering the breakdown of the cheese in the mouth during consumption and affecting the rate of release of volatile compounds that contributes to flavour [37,39]. Furthermore, proteolysis can substantially alter cheese protein composition during the ripening period, changing the binding ability of volatile compounds and influencing their release into the mouth, resembling the process previously described for fat [37].

Hence, the changes induced by cardoon type in the chemical characteristics of the cheese may affect texture, flavour and aroma and, therefore, be accepted by a wider diversity of consumers.

### 3.2.2. Influence of Cheese Production Month

The production month significantly affected rind colour coordinates a* and b*, allowing to distinguish cheeses produced in each one of the studied months, as shown in Table 4. Concerning the blue–yellow component (b*), there was not a clear tendency through the milking season, with cheeses produced in February yellower than in the remaining production dates. Concerning lightness (L*), cheeses produced in February and April stood out as the ones with a lighter colour. The values shown in Table 4 are similar to those reported in previous research [3,40].

Contrary to what happens in cheese rind, there are a tendency for cheese paste to acquire a greater balance between green–red tone as the milking season proceeds, while the yellow colour gradually fades from November to April. Still, there are no significant differences between cheeses produced in November and February when comparing paste colour.

The texture of PDO SE cheese has been extensively studied by other authors [3,18,41,42]; however, as far as is known, this is the first conducted study that takes into consideration the effect of different production months. The analysis performed showed that cheese production month has a highly significant influence (*p* ≤ 0.001) on rind texture attributes. All cheese texture attributes showed a progressive decrease during the milking season. Regarding hardness, November stood out as the month where cheeses were harder, with values about four times higher than in the subsequent months.

Comparing cheese chemical parameters, significant main effects were detected for fat and protein. As the milking season progresses, an increase in fat content and a decrease in protein content were evident. The increase in fat content is consistent with the results obtained for milk shown in Table 1. With respect to protein, the decrease verified in milk during the milking season was also observed in cheese characteristics. The mean values obtained for fat and protein content, presented in Table 4, are in agreement with the data reported in a previous study comprising PDO SE cheese [43], although the results obtained are superior than most publications [44,45,46].

As previously reported, cheese chemical composition has a major influence in cheese texture. Considering the variance observed in fat and protein content during the milking season, the changes observed in rind texture attributes were to be expected. Additionally, the relationship observed between fat content and texture during the season was in accordance with the results obtained in previous studies. As fat content increases, cheeses show lower values for hardness and springiness [47,48].

### 3.2.3. Variation of Physical and Chemical Parameters According to Cardoon Type during the Flock Lactation Stage

In addition to the main effects previously discussed, the possible occurrence of interactions between cardoon type and production month must be considered. For example, in attributes such as rind colour coordinates L* and ∆E, cohesiveness and resilience, the cardoon type by itself does not have a significant influence (Table 2). However, for the same attributes, the interaction effect between cardoon type and production month is significant. This means that the influence of cardoon type on the characteristics of the cheese may depend on the month of production and vice-versa.

**Figure 1:**
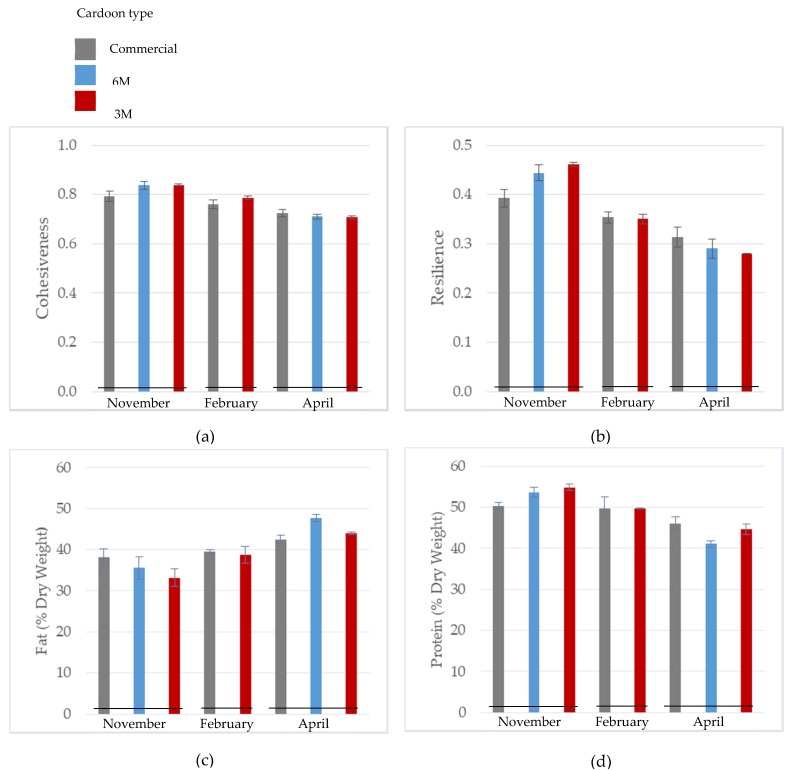
Variation of physical and chemical parameters: (**a**) cohesiveness, (**b**) resilience, (**c**) fat and (**d**) protein in PDO SE cheese according to cardoon type during the flock lactation stage.

In the following sections, the results where the interactions are very significant (*p* < 0.01) will be explored, namely rind cohesiveness, rind resilience, fat and protein content (Table 2). Figure 1 shows the segregated results for the parameters listed above, according to each cardoon type during the milking season, thus allowing us to examine clearly the interactions that occurred between the main effects studied.

In November and February, the use of the commercial cardoon promoted a reduction in rind cohesiveness values, while 3 M cardoon showed an inverse trend. However, this tendency was reversed at the end of the milking season, showing that the cardoon type effect in cohesiveness depends on the cheese production month. The same tendency was found for rind resilience, agreeing with the results shown in Table 4 concerning the interaction effect significance.

Regarding the chemical attributes, displayed in Figure 1, cheese produced with commercial cardoon tends to maintain the fat content constant throughout the milking season. On the other hand, cheeses produced with 6 M and 3 M cardoon types tend to gradually increase the fat percentage during the flock lactation stage. When considering the results obtained regarding protein content, it stands out that its behaviour for each cardoon type during the milking season is the opposite to that observed for fat content.

February stands out as the time of the year in which each attribute shown in Figure 1 present the closest values for the distinct cardoon types studied. These results suggest that Serra da Estrela cheeses produced in February, at the mid-point of the milking season, are likely to have similar characteristics in terms of texture and chemical parameters, regardless of the cardoon type used in their production.

These results show that the cardoon flower can be used in cheese factories to achieve a final product with the desired characteristics in the attributes described above, when used in accordance to the production month.

## 4. Conclusions

This study showed that cardoon flower type affects, mainly, fat and rind colour parameters; however, it shows no significant influence on cheese texture. Concerning chemical properties, 6 M cardoon produced cheeses with the highest fat content, while those produced with 3 M cardoon showed the highest protein percentage. This data suggests that, even though the relative quantity of cardoon used in PDO SE cheese is very small, it may have a decisive influence on its physicochemical characteristics.

Concerning Serra da Estela sheep lactation stage, the results of this study suggest that it has a significant impact on milk and cheese composition. Early stages of the ewe’s lactation produced cheeses richer in protein and poorer in fat. Physical characteristics, namely rind colour, rind texture and paste colour, were also affected by the ewe’s lactation stage. The cheeses produced in an early stage of lactation were harder, with a yellower paste colour.

The understanding of the interactions that occur between the influence of cardoon type and the flock lactation stage can help to explain the different behaviour of cheese produced with each cardoon type, according to production month. This may allow cheese factories to regulate cheese production in order to obtain a final product with the desired characteristics.

This study concludes that the seasonal changes have a considerable impact in PDO SE cheese physical and chemical attributes. Although the cardoon type seems to have a more restrained effect, when used with expertise, it can help adjust sensory characteristics of the cheese in order to obtain a final product that matches consumer acceptability requirements.

## Figures and Tables

**Table 1:** Characteristics of the milk used in the production of PDO Serra da Estrela cheese.

Milk Characteristics	Production Month		
	November	February	April
Fat DW^1^ (%)	30.69 ± 0.10 ^aꝉ^	44.91 ± 0.19 ^b^	41.57 ± 0.05 ^c^
Protein DW^1^ (%)	32.57 ± 0.27 ^a^	31.10 ± 0.24 ^b^	30.98 ± 0.07 ^b^
Dry matter (%)	16.30 ± 0.03 ^a^	16.44 ± 0.01 ^b^	20.03 ± 0.03 ^c^
Acidity (cm^3^ NaOH/ dm^3^)	28.50 ± 0.71 ^a^	26.33 ± 0.58 ^a^	29.33 ± 2.08 ^a^

^1^ DW-Dry Weight; ꝉ Different letters indicate significant differences between groups (p < 0.05).

**Table 2:** Mean square percentages and statistical significance of cheese production month, cardoon type and their interaction. The level of significance is obtained by an ANOVA unifactorial model.

Attributes		Mean Squares (%)				Level of Significance		
		Production Month (M)	Cardoon Type (C)	Interaction (C^x^M)	Error	M	C	M^x^C
Rind colour	L*	76.0	5.4	14.4	4.2	***	n.s.	*
	a*	87.6	9.3	2.0	1.1	***	**	n.s.
	b*	88.8	7.1	3.4	0.7	***	**	*
	∆E	85.4	0.1	11.1	3.4	***	n.s.	*
Paste colour	L*	2.3	31.9	22.2	43.6	n.s.	n.s.	n.s.
	a*	77.6	12.0	8.4	2.0	***	*	*
	b*	93.0	3.6	0.5	2.9	***	n.s.	n.s.
	∆E	91.9	0.5	4.6	3.0	***	n.s.	n.s.
Rind Texture	Hardness (N)	95.1	1.9	2.1	0.9	***	n.s.	n.s.
	Springiness (N.s)	94.1	2.8	2.4	0.8	***	n.s.	n.s.
	Cohesiveness	91.5	2.7	4.9	0.9	***	n.s.	**
	Resilience	92.5	0.7	6.4	0.4	***	n.s.	***
Fat (DW)		85.0	6.4	7.9	0.7	***	**	***
Protein (DW)		87.0	3.6	8.6	0.7	***	*	***
Salt (DW)		22.2	31.7	33.2	12.9	n.s.	n.s.	n.s.
Moisture (%)		75.4	3.7	18.2	2.8	***	n.s.	**
Weight loss (%)		94.5	0.6	1.3	3.5	***	n.s.	n.s.
pH		18.8	7.4	53.6	20.3	n.s.	n.s.	n.s.

* *p* < 0.05; ** *p* < 0.01;*** *p* < 0.001; n.s. no significant differences.

**Table 3:** The influence of cardoon flower type (commercial, 6 M and 3 M) in the physicochemical attributes of cheeses, accessed by analysis of variance. SD-standard deviation

Attributes		Cardoon Type		
		Commercial	6 M	3 M
		Mean SD	Mean SD	Mean SD
Rind Colour	L*	63.13 ± 3.20 ^a ꝉ^	62.49 ± 2.70 ^a^	62.36 ± 1.31 ^a^
	a*	−2.04 ± 0.55 ^a^	−2.38 ± 0.44 ^b^	−2.37 ± 0.40 ^b^
	b*	22.06 ± 2.60 ^a^	20.80 ± 0.84 ^b^	21.23 ± 2.16 ^b^
	∆E	39.31 ± 3.20 ^a^	39.16 ± 2.68 ^a^	39.51 ± 1.13 ^a^
Paste Colour	L*	80.11 ± 1.55 ^a^	81.98 ± 2.22 ^a^	79.94 ± 2.05 ^a^
	a*	−4.11 ± 1.00 ^a^	−3.69 ± 1.50 ^b^	−4.35 ± 1.19 ^a^
	b*	18.33 ± 4.70 ^a^	16.05 ± 5.50 ^a^	17.81 ± 4.59 ^a^
	∆E	24.26 ± 2.74 ^a^	21.08 ± 4.87 ^a^	23.81 ± 3.74 ^a^
Rind Texture	Hardness (N)	2.09 ± 1.27 ^a^	3.25 ± 2.33 ^a^	2.80 ± 2.16 ^a^
	Springiness (N.s)	0.82 ± 0.04 ^a^	0.83 ± 0.07 ^a^	0.83 ± 0.06 ^a^
	Cohesiveness	0.76 ± 0.03 ^a^	0.77 ± 0.07 ^a^	0.78 ± 0.06 ^a^
	Resilience	0.35 ± 0.04 ^a^	0.37 ± 0.09 ^a^	0.36 ± 0.08 ^a^
Fat (DW)		40.04 ± 2.12 ^a^	41.67 ± 6.81 ^b^	38.64 ± 4.79 ^a^
Protein (DW)		48.68 ± 2.31 ^a,b^	47.35 ± 7.04 ^a^	49.76 ± 4.50 ^b^
Salt (DW)		3.40 ± 0.35 ^a^	3.48 ± 0.20 ^a^	3.68 ± 0.41 ^a^
Moisture (%)		51.9 ± 1.0 ^a^	51.1 ± 2.5 ^a^	52.2 ± 2.1 ^a^
Weight loss (%)		−28.00 ± 4.13 ^a^	−28.70 ± 4.75 ^a^	−27.40 ± 3.44 ^a^
pH		5.29 ± 0.13 ^a^	5.26 ± 0.09 ^a^	5.25 ± 0.14 ^a^

^ꝉ^ Different letters indicate significant differences between groups (*p* < 0.05).

**Table 4:** The influence of flock lactation stage (November, February and April) in the physicochemical attributes of cheeses, accessed by analysis of variance. SD-standard deviation

Attributes		Cheese production Month		
		November	February	April
		Mean SD	Mean SD	Mean SD
Rind colour	L*	60.72 ± 1.39 ^aꝉ^	64.07 ± 0.70 ^b^	64.35 ± 2.45 ^b^
	a*	−1.84 ± 0.27 ^a^	−2.24 ± 0.23 ^b^	−2.80 ± 0.16 ^c^
	b*	21.28 ± 0.87 ^a^	24.68 ± 0.68 ^b^	19.44 ± 0.71 ^c^
	∆E	40.90 ± 1.53 ^a^	39.83 ± 0.43 ^a^	36.94 ± 2.19 ^b^
Paste colour	L*	80.84 ± 0.58 ^a^	79.60 ± 2.27 ^a^	80.86 ± 2.41 ^a^
	a*	−5.13 ± 0.33 ^a^	−4.99 ± 0.25 ^a^	−2.82 ± 0.27 ^b^
	b*	21.83 ± 0.62 ^a^	21.17 ± 0.64 ^a^	12.38 ± 0.92 ^b^
	∆E	26.07 ± 0.63 ^a^	26.37 ± 1.08 ^a^	19.48 ± 2.17 ^b^
Rind Texture	Hardness (N)	4.92 ± 0.95 ^a^	1.56 ± 0.19 ^b^	1.10 ± 0.14 ^c^
	Springiness (N.s)	0.88 ± 0.02 ^a^	0.84 ± 0.01 ^b^	0.77 ± 0.02 ^c^
	Cohesiveness	0.82 ± 0.03 ^a^	0.77 ± 0.02 ^b^	0.71 ± 0.01 ^c^
	Resilience	0.43 ± 0.03 ^a^	0.35 ± 0.01 ^b^	0.29 ± 0.02 ^c^
Fat (DW)		35.62 ± 2.55 ^a^	39.12 ± 1.06 ^b^	44.76 ± 2.40 ^c^
Protein (DW)		52.96 ± 2.35 ^a^	49.75 ± 1.16 ^b^	43.89 ± 2.35 ^c^
Salt (DW)		3.62 ± 0.44 ^a^	3.36 ± 0.38 ^a,b^	3.54 ± 0.24 ^a^
Moisture (%)		53.1 ± 1.4 ^a^	52.5 ± 0.9 ^a^	50.0 ± 1.2 ^b^
Weight loss (%)		−32.21 ± 1.71 ^a^	−26.05 ± 1.49 ^b^	−24.96 ± 2.51 ^b^
pH		5.25 ± 0.12 ^a^	5.23 ± 0.18 ^b^	5.30 ± 0.08 ^a^

^ꝉ^ Different letters indicate significant differences between groups (*p* < 0.05); The asterisk (*) after L, a and b is part of the colour coordinates full name. It is a way to distinguish them from Hunter Lab color space coordinates (L, a and b).
